# Secretory leukocyte protease inhibitor protects against severe urinary tract infection in mice

**DOI:** 10.1128/mbio.02554-23

**Published:** 2024-01-25

**Authors:** Anne L. Rosen, Michael A. Lint, Dayne H. Voelker, Nicole M. Gilbert, Christopher P. Tomera, Jesús Santiago-Borges, Meghan A. Wallace, Thomas J. Hannan, Carey-Ann D. Burnham, Scott J. Hultgren, Andrew L. Kau

**Affiliations:** 1 Division of Allergy and Immunology, Department of Medicine, Washington University School of Medicine, St. Louis, Missouri, USA; 2 Center for Women’s Infectious Disease Research, Washington University School of Medicine, St. Louis, Missouri, USA; 3 Division of Infectious Diseases, Department of Pediatrics, Washington University School of Medicine, St. Louis, Missouri, USA; 4 Department of Pathology and Immunology, Washington University School of Medicine, St. Louis, Missouri, USA; 5 Department of Molecular Microbiology, Washington University School of Medicine, St. Louis, Missouri, USA; 6 Department of Medicine, Washington University School of Medicine, St. Louis, Missouri, USA; University of Michigan-Ann Arbor, Ann Arbor, Michigan, USA

**Keywords:** uropathogenic *Escherichia coli* (UPEC), secretory leukocyte protease inhibitor (SLPI), urinary tract infection (UTI), innate immunity, antimicrobial peptide (AMP), protease inhibitor, neutrophil elastase (NE), neutrophil, inflammation, mouse model, SLPI knockout, human study

## Abstract

Millions suffer from urinary tract infections (UTIs) worldwide every year with women accounting for the majority of cases. Uropathogenic *Escherichia coli* (UPEC) causes most of these primary infections and leads to 25% becoming recurrent or chronic. To repel invading pathogens, the urinary tract mounts a vigorous innate immune response that includes the secretion of antimicrobial peptides (AMPs), rapid recruitment of phagocytes, and exfoliation of superficial umbrella cells. Here, we investigate secretory leukocyte protease inhibitor (SLPI), an AMP with antiprotease, antimicrobial, and immunomodulatory functions, known to play protective roles at other mucosal sites, but not well characterized in UTIs. Using a preclinical model of UPEC-caused UTI, we show that urine SLPI increases in infected mice and that SLPI is localized to bladder epithelial cells. UPEC-infected SLPI-deficient (*Slpi^−/−^*) mice suffer from higher urine bacterial burdens, prolonged bladder inflammation, and elevated urine neutrophil elastase (NE) levels compared to wild-type (*Slpi^+/+^*) controls. Combined with bulk bladder RNA sequencing, our data indicate that *Slpi^−/−^* mice have a dysregulated immune and tissue repair response following UTI. We also measure SLPI in urine samples from a small group of female subjects 18–49 years old and find that SLPI tends to be higher in the presence of a uropathogen, except in patients with a history of recent or recurrent UTI, suggesting a dysregulation of SLPI expression in these women. Taken together, our findings show SLPI promotes clearance of UPEC in mice and provides preliminary evidence that SLPI is likewise regulated in response to uropathogen exposure in women.

**IMPORTANCE:**

Annually, millions of people suffer from urinary tract infections (UTIs) and more than $3 billion are spent on work absences and treatment of these patients. While the early response to UTI is known to be important in combating urinary pathogens, knowledge of host factors that help curb infection is still limited. Here, we use a preclinical model of UTI to study secretory leukocyte protease inhibitor (SLPI), an antimicrobial protein, to determine how it protects the bladder against infection. We find that SLPI is increased during UTI, accelerates the clearance of bacteriuria, and upregulates genes and pathways needed to fight an infection while preventing prolonged bladder inflammation. In a small clinical study, we show SLPI is readily detectable in human urine and is associated with the presence of a uropathogen in patients without a previous history of UTI, suggesting SLPI may play an important role in protecting from bacterial cystitis.

## INTRODUCTION

In the United States, urinary tract infections (UTIs) lead to over 10 million office visits and cost more than $3 billion annually in sick leave and treatment (1, 2). Women are at higher risk of developing frequent UTIs than men (3–5) and the primary culprit of UTIs is uropathogenic *Escherichia coli* (UPEC), a Gram-negative bacterium responsible for most community-acquired infections (6, 7). UPEC strains are common gut commensals and do not cause disease in the gastrointestinal (GI) tract, but once shed, they can colonize the periurethral tissues (6, 8). UPEC is known to utilize multiple virulence factors to aid its ascent, adherence, and invasion of the bladder epithelium (9). Among these virulence factors, type 1 pili, a hair-like extracellular structure, are essential for adhesion and invasion of UPEC to the luminal superficial facet epithelial cells of the bladder (7, 10, 11). Once inside the bladder epithelium, UPEC can escape the immune response by replicating within the facet cells to form intracellular bacterial communities (IBCs) that evade phagocytosis by neutrophils (12–14). In response to infection, superficial epithelial cells undergo a process similar to apoptosis and are exfoliated into the urine (10, 15).

While the adaptive immune system responds to UPEC-caused UTI, the innate immune response is thought to play a key role in controlling infection (16). Toll-like receptors (TLRs) on host cells recognize conserved molecules like LPS (17) and flagellin (18) expressed on the surface of Gram-negative bacteria like UPEC. Once TLR4 recognizes LPS, the NF-κB pathway becomes activated resulting in the transcription of inflammatory cytokines including IL-1β, IL-6, and TNFα (19, 20). Accumulation of these molecules leads to the recruitment of neutrophils which is crucial in controlling early infection with UPEC (20–22). However, the recruitment of neutrophils is carefully regulated to maintain a balance necessary to clear infection while protecting host tissues. An inadequate number of neutrophils can result in failure to clear UPEC from the bladder, but an overabundance of neutrophils can lead to chronic cystitis and excessive tissue damage (23, 24), emphasizing the delicate homeostasis that must be maintained to resolve UTI.

Increased levels of proinflammatory cytokines also upregulate expression of antimicrobial peptides (AMPs), which help prevent invasive bacterial infections (25, 26). AMPs are small, positively charged proteins that are commonly produced by neutrophils, macrophages, and epithelial cells as well as other cell types. While they can be constitutively expressed, production of AMPs is also induced by microbial stimuli and tissue damage (27, 28). While AMPs including cathelicidin (29–31), defensins (32–35), ribonucleases (36–39), and serpins (24), have been studied in the context of UTI, little is known about the roles and functions of the well-characterized AMP secretory leukocyte protease inhibitor (SLPI) which is also found in the urogenital tract (40, 41). SLPI has been extensively studied in the respiratory tract (42), but has also been shown to help combat urogenital infections from HIV, gonorrhea, and *Candida* yeast (43–45), though its function in bacterial UTIs remains unknown. SLPI is a small, cationic protein containing two domains that demonstrate independent functions (46–49). The N-terminal domain is associated with antimicrobial activity and has been reported to demonstrate bactericidal activity against *E. coli* and *Staphylococcus aureus in vitro* (47). The C-terminal domain broadly inhibits serine proteases and may be important in limiting inflammation caused by enzymes, including those released by activated neutrophils such as cathepsin G and neutrophil elastase (NE) (24, 48, 50). NE, a serine protease stored in preformed granules (51), aids the immune system by digesting phagocytosed proteins (52). NE released from activated neutrophils can also degrade extracellular matrix proteins like elastin (52, 53) and fibronectin (54) resulting in host tissue damage (52, 54–56). Prior reports show SLPI can directly inhibit NE protease function and reduce damage in response to inflammation (28, 57). Additionally, SLPI has been shown to regulate the immune response through the direct blocking of the NF-κB promoter and by preventing the degradation of NF-κB inhibitors (58–60). Other serine protease inhibitors from the serpin family are also increased during UTI (24).

In this study, we tested the hypothesis that SLPI protects the bladder from UPEC-caused cystitis using a preclinical model of UTI. We found that mice respond to the introduction of UPEC in the urinary tract by upregulating SLPI within hours of infection. We also found that *Slpi^−/−^* mice demonstrate evidence of a dysregulated immune response to UPEC infection, while also exhibiting an increased burden of UPEC in the urine and delayed resolution of inflammation at later time points. We complement these findings in mice with a small clinical study showing that women with acute bacteriuria tend to have elevated SLPI levels, providing preliminary evidence that SLPI plays a protective role in human UTIs.

## RESULTS

### SLPI is increased in the mouse urinary tract following UTI

**Fig 1:**
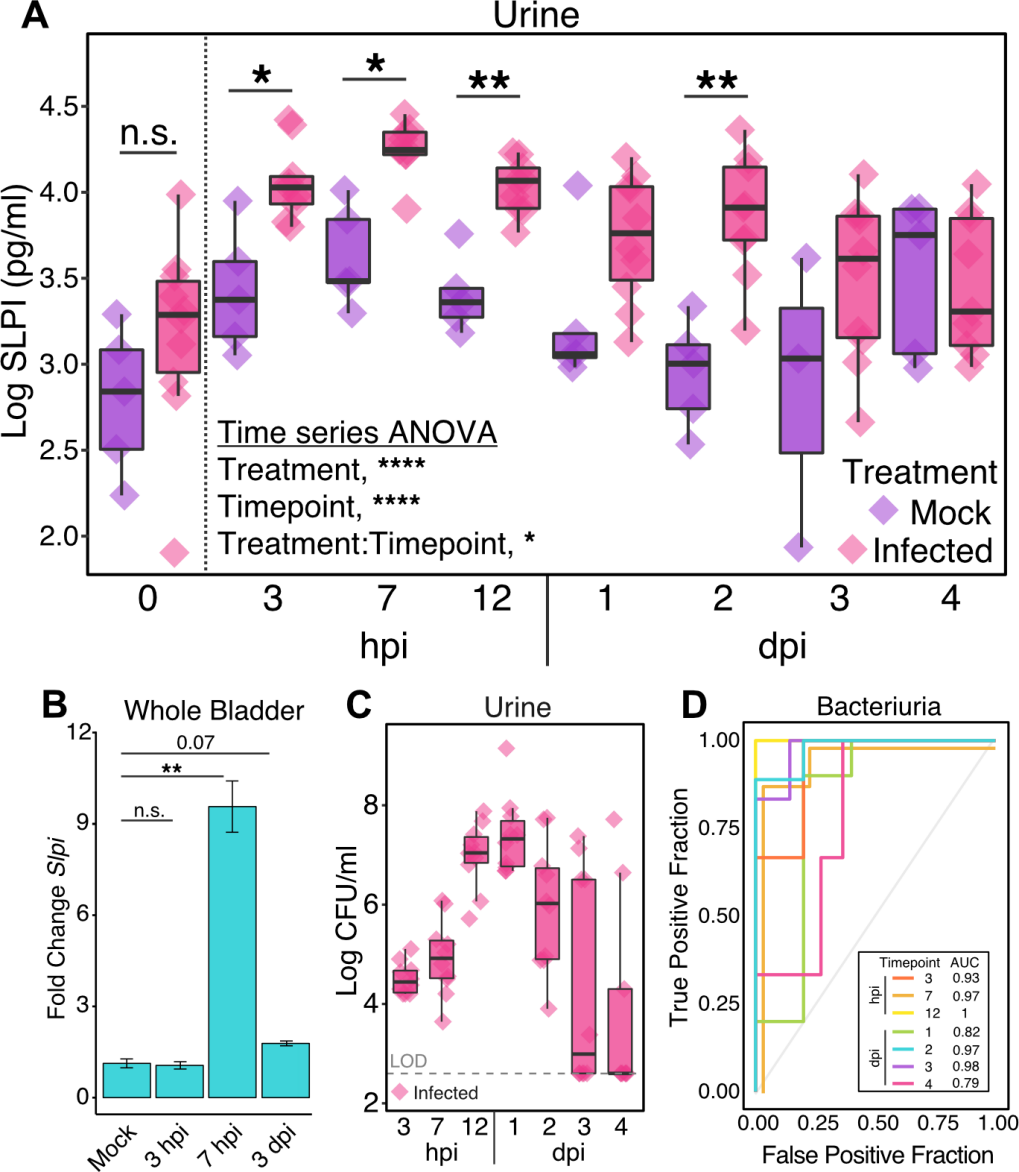
SLPI is increased in mouse urinary tract following UTI. (**A**) Urine SLPI levels in C57BL/6J mock-infected (*n* = 3–5) and UTI89-infected (*n* = 9–10) mice following log base 10 transformation. A time series ANOVA was performed on all time points after 0 including imputed values for missing samples (two mock and five infected). A post hoc Student’s *t* test was performed on imputed data with FDR correction. (**B**) qRT-PCR of *Slpi* in whole bladder homogenates at 3 hpi (*n* = 12), 7 hpi (*n* = 8), and 3 dpi (*n* = 3) represented as fold change to mock-infected mice (*n* = 3–4) (Student’s *t* test). (**C**) Log base 10 CFU/mL of UTI89 in urine of infected mice over time. Dashed line represents limit of detection (LOD). (**D**) ROC curve analysis using SLPI levels in panel **A** to classify bacteriuria shown in panel **C** with area under the curve (AUC) values listed for each timepoint. Time points 7 hpi and 2 dpi have been offset slightly to aid visualization.

We used an established mouse model of UTI to determine whether SLPI levels are increased in the urine following infection (61). Briefly, we grew a kanamycin-marked strain of UTI89, a prototypical UPEC clinical isolate (12), under static conditions to induce expression of type 1 pili (11, 62) before transurethrally inoculating ~1 × 10^8^ CFU or sterile phosphate-buffered saline (PBS) directly into the bladders of C57BL/6J female mice. Urine and tissues were collected at multiple time points for further analysis. We first measured the concentration of SLPI in urine by ELISA and found a significant increase (*P* = 0.02) in infected mice compared to mock-infected mice at 3 hours post-infection (hpi). This concentration peaked at 7 hpi before slowly decreasing over a 4-day period (Fig. 1A). A time series ANOVA showed that infected mice had significantly higher levels of SLPI over the course of infection compared to mock-infected controls (Treatment, *P* = 1.5 × 10^−6^). Additionally, transcription of *Slpi* was increased in bladder homogenates 7 hpi, suggesting that SLPI protein found in the urine is originating, at least in part, from the bladder and not solely as increased urinary secretion of SLPI from the kidneys (*P* = 0.001; Fig. 1B). Interestingly, bacteriuria (UPEC in the urine) peaked 1 day after infection (Fig. 1C), long after the apex of urine SLPI levels, suggesting that SLPI is involved in the early stages of infection. To determine if urine SLPI levels are associated with bacteriuria, we created a receiver operating characteristic (ROC) curve for each time point. Curves with a greater area under the curve (AUC) indicate improved performance of SLPI as a predictor of bacteriuria, while AUC values less than 0.5 (the gray line) indicate the prediction is no better than chance. These ROC curves show that the amount of SLPI is associated with bacteriuria in mice at each time point we examined from 3 hpi to 4 days post-infection (dpi) with an AUC value of 0.79 or greater (Fig. 1D).

### SLPI is expressed in the bladder epithelium

**Fig 2:**
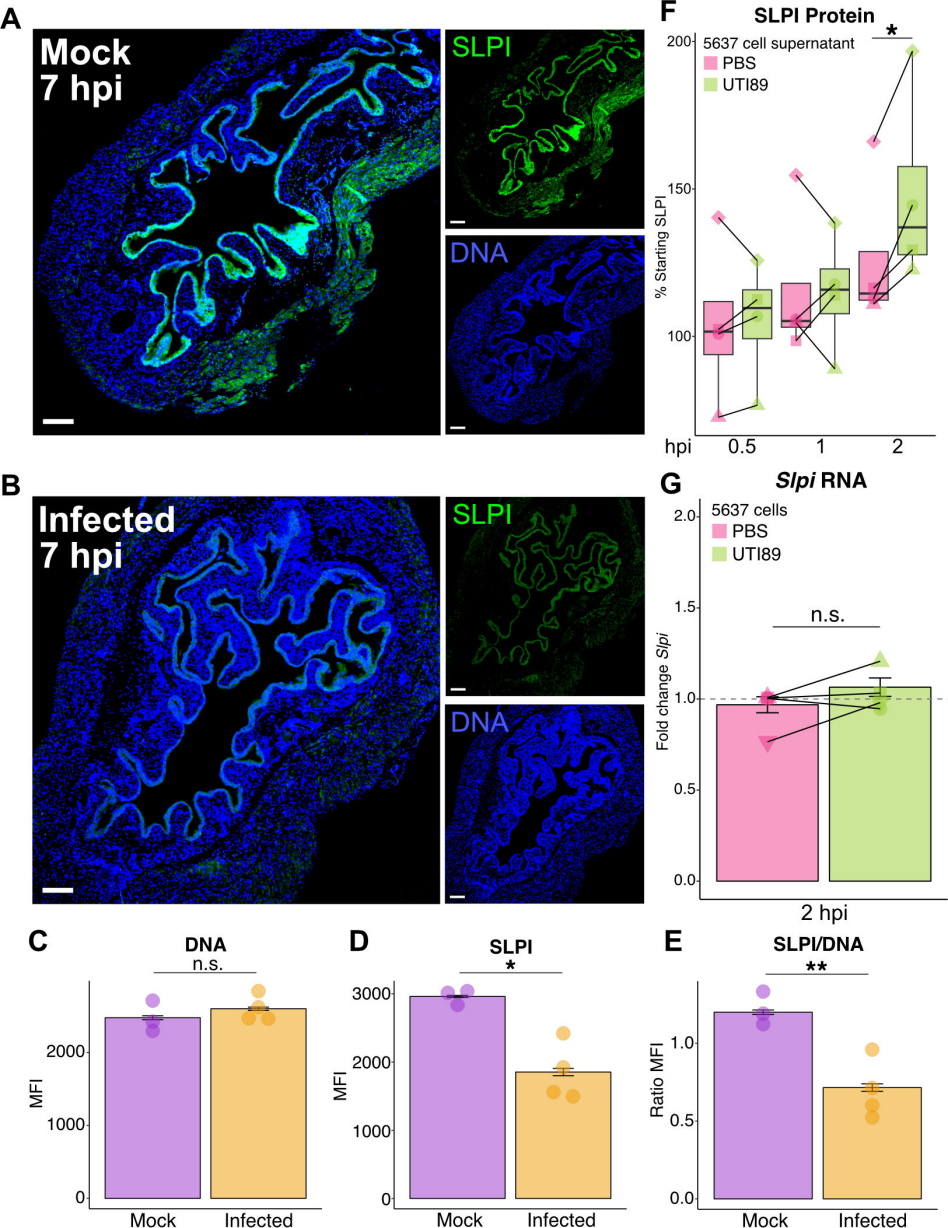
SLPI is expressed in the bladder epithelium. (**A and B**) Immunofluorescence staining of SLPI protein expression (green) and DNA using Hoechst stain (blue) in mock-infected (**A**) and infected (**B**) bladders from *Slpi^+/+^* mice. Left panels (5× magnification, 200 µm scale bars) are merged images of SLPI and DNA shown as smaller panels on the right. (**C–E**) Mean fluorescence intensity (MFI) of mock and infected bladder epithelium for DNA (**C**) and SLPI (**D**) with MFI ratio of SLPI/DNA (**E**) (Student’s *t* test). (**F**) Comparison of supernatant SLPI levels in control (PBS) or infected (UTI89) 5637 bladder epithelial cells represented as percent of starting SLPI in each well. (**G**) Fold change in *Slpi* transcription as measured by qRT-PCR in 5637 cells at 2 hpi. For panels **F** and **G**, shapes denote separate biological replicate experiments, each point represents the average of 4 or more individual wells (paired Student’s *t* test).

To better understand localization of SLPI expression in the urinary tract, we collected bladders from *Slpi^+/+^* and *Slpi^−/−^* mice 7 h after infection and performed immunofluorescent staining against SLPI protein (Fig. 2) along with isotype controls (Fig. S1). Staining with an SLPI-specific antibody revealed a strong signal on the luminal surface of the bladder, consistent with staining of the uroepithelium in both the mock-infected and UTI89-infected mice (Fig. 2A and B). Control staining with an isotype antibody (Fig. S1A through C) or against *Slpi^−/−^* mouse bladders (Fig. S1D) failed to produce noticeable signal, indicating that the observed staining was specific to SLPI. While we did observe some staining of SLPI in the tissues underlying the epithelium, the fluorescence was less prominent than the signal observed in the uroepithelium, implying that SLPI is primarily expressed by the epithelium in the bladder. Next, we quantified the difference in SLPI fluorescence of the uroepithelium between mock and infected mice. While there was no statistically significant difference in DNA fluorescence (Fig. 2C), we did see a decreased SLPI signal in infected mice both before (*P* = 0.01; Fig. 2D) and after normalizing to DNA fluorescence (*P* = 0.009; Fig. 2E). This finding implies that SLPI protein is largely depleted from the bladder epithelium 7 hpi either through exfoliation of bladder epithelial cells or secretion, while transcription of SLPI is upregulated (Fig. 1B). To better understand the dynamics between SLPI transcription and secretion, we examined the human bladder epithelial cell line, 5637, to determine the epithelial response of SLPI to UPEC exposure. We found that infection with UTI89 resulted in significantly increased secretion of SLPI protein into the culture supernatant within 2 h of infection compared to cells exposed to PBS alone (*P* = 0.02; Fig. 2F). However, the increases in SLPI protein apparent in the culture supernatant were not accompanied by increases in cellular transcripts of SLPI at that time point (Fig. 2G), suggesting that increases in SLPI quantity in the urinary tract may not be transcriptionally mediated in the period immediately after UTI89 exposure.

### *Slpi^−/−^* mice have prolonged bacteriuria compared to *Slpi^+/+^* mice

**Fig 3:**
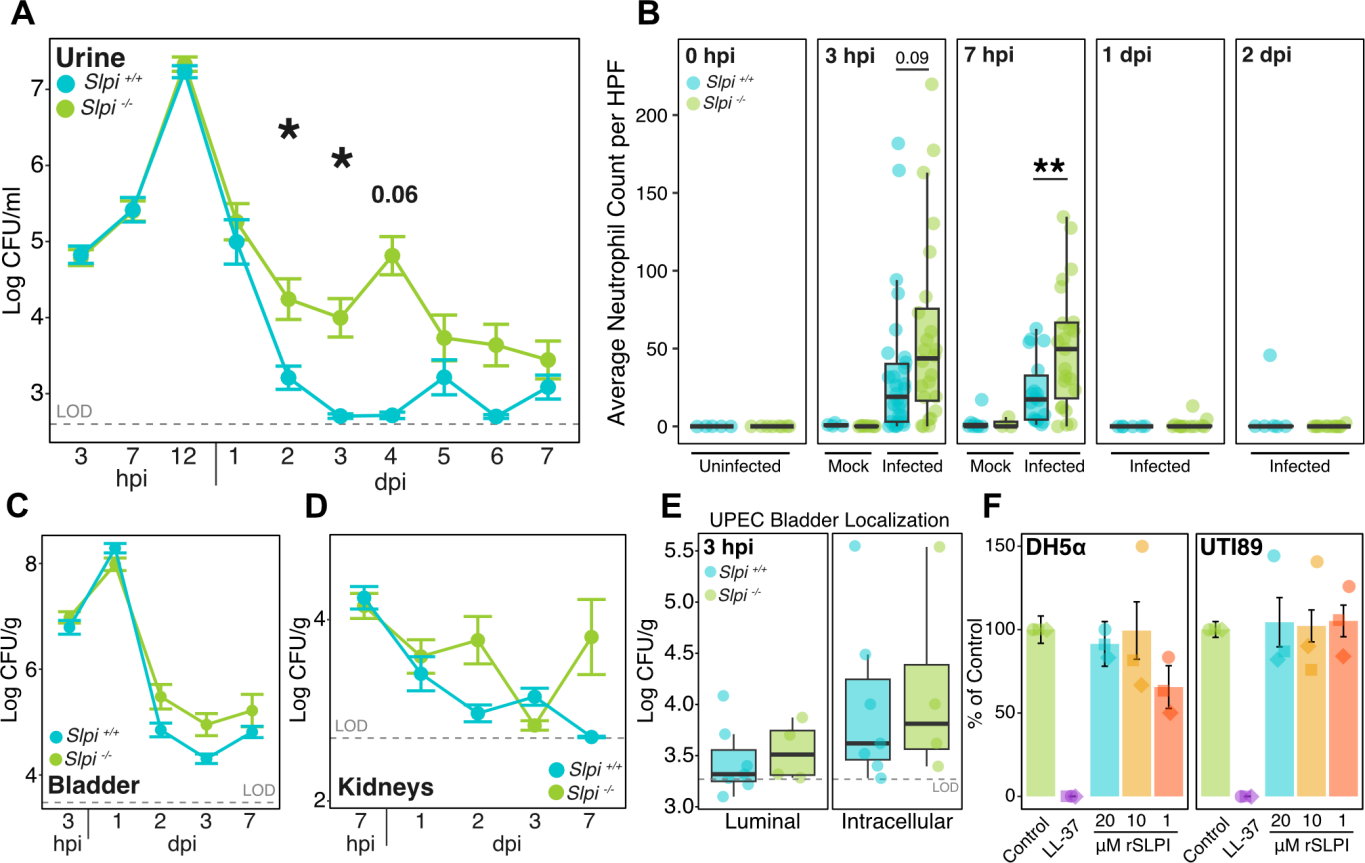
*Slpi^−/−^* mice have prolonged bacteriuria compared to *Slpi^+/+^* mice. (**A**) Urine bacterial titers from *Slpi^+/+^* (blue) and *Slpi^−/−^* mice (green) during 1 week of infection with UTI89. Data are log base 10 transformed CFU/mL and are combined from seven experiments with 5–37 mice per genotype per time point (Student’s *t* test). (**B**) Averaged cytospin cell counts of neutrophils in urine from uninfected (*n* = 5–8), mock-infected (*n* = 3–8), and UTI89-infected (*n* = 6–26) *Slpi^+/+^* (blue) and *Slpi^−/^*^−^ mice (green) from 0 hpi to 2 dpi. Data are combined from four separate experiments (Student’s *t* test). (**C**) Bladder bacterial titers in log base 10 CFU/g. Data are combined from four experiments with 5–14 mice per genotype per time point. (**D**) Kidney bacterial titers in log base 10 CFU/g. Data are combined from seven experiments with 5–19 mice per genotype per time point. (**E**) *Ex vivo* gentamicin protection assay on infected bladders from *Slpi^+/+^* (blue) and *Slpi^−/−^* mice (green) showing log CFU/g of tissue for recovered UTI89 from luminal or intracellular samples. (**F**) *In vitro* recombinant SLPI (rSLPI) antimicrobial activity against UTI89 and DH5α in PBS + 1% LB. All samples were normalized to PBS control group (green) and 1 µM LL-37 antimicrobial protein was used as a positive control (purple). The final concentrations of rSLPI in each culture were: 20 µM (blue), 10 µM (yellow), and 1 µM (orange). For panels **A–E**, gray dashed lines on each plot represent average limit of detection (LOD) for that tissue type.

To investigate whether SLPI is involved in susceptibility to UPEC-caused UTI, we utilized 129;BL/6 SLPI^tm1Smw^/J mice (28), which are engineered to lack SLPI, and back-crossed them onto a C57BL/6J background for at least 10 generations (*Slpi^−/−^* mice). After infecting mice with UTI89, we found that compared to wild-type (*Slpi^+/+^*) mice, *Slpi^−/−^* mice had significantly more bacteria in their urine at 2 (*P* = 0.02) and 3 dpi (*P* = 0.02; Fig. 3A). We also measured the abundance of neutrophils in urine sediments (pyuria) following infection with UTI89 and found that they were significantly increased in *Slpi^−/−^* mice compared to *Slpi^+/+^* mice 7 hours after infection (*P* = 0.006) but returned to *Slpi^+/+^* levels by 24 hpi (Fig. 3B). The increased urine neutrophils did not correspond to differences in urine titers at this time point (Fig. 3A) suggesting that a lack of SLPI may lead to more pronounced early neutrophil recruitment that does not impact bacterial titers. We also did not see any statistically significant differences in bladder or kidney bacterial burdens over the 7-day UTI model (Fig. 3C and D; see also Fig. S2A through C). We next used an *ex vivo* gentamicin protection assay (63) to test whether UTI89 invasion into bladder cells was altered in *Slpi^−/−^* mice but found no statistically significant differences from *Slpi^+/+^* (Fig. 3E). Taken together, we observed that the time point where urine SLPI is first increased does not coincide with reduced UTI89 urine titers, suggesting that SLPI is not directly inhibiting UTI89 growth in our model.

Next, we wanted to test if SLPI could inhibit the growth of UTI89 *in vitro*. Using an approach based on previously described protocols (47, 64), we tested if exposure to a commercially available recombinant SLPI (rSLPI) protein could reduce the viability of UTI89. We found that even after supraphysiological exposures to rSLPI, the presence of rSLPI did not significantly reduce the viability of UTI89 or a lab strain of *E. coli* (DH5α) (Fig. 3F). In contrast, exposure to LL-37, the processed form of human cathelicidin, is known to kill *E. coli* (65), and readily reduced the viability of both UTI89 and DH5α in the same assay. These results show that SLPI does not exert strong antimicrobial activity against *E. coli* and supports the idea that other functions of SLPI likely mediate its effect on UTI in our preclinical model.

### *Slpi^−/−^* mice demonstrate a dysregulated immune response in the bladder at 1 dpi

**Fig 4:**
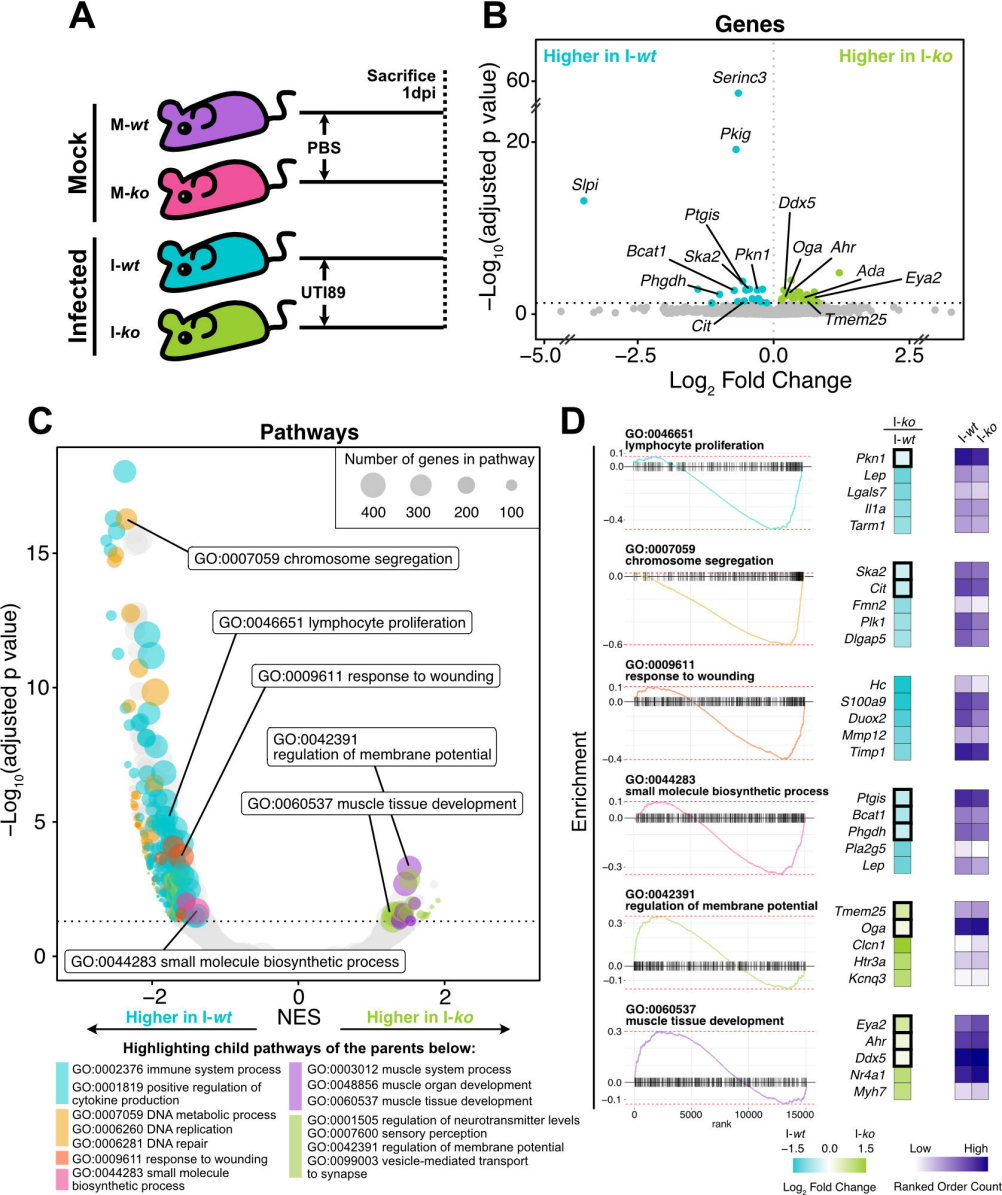
*Slpi^−/−^* mice demonstrate a dysregulated immune response in the bladder at 1 dpi. (**A**) Schematic of experimental approach to assess differences in whole bladder transcriptome of mock-infected *Slpi^+/+^* (M-*wt*, *n* = 4) and *Slpi^−/−^* (M-*ko*, *n* = 3) and their UTI89-infected *Slpi^+/+^* (I-*wt*, *n* = 14) and *Slpi^−/−^* (I-*ko*, *n* = 11) counterparts. (**B**) Volcano plot showing genes significantly upregulated in infected *Slpi^+/+^* (I-*wt*, blue) and infected *Slpi^−/−^* mice (I-*ko*, green) above the dotted line. (**C**) Bubble volcano plot showing gene set enrichment analysis of GO pathways between I-*wt* and I-*ko* mice. Differentially expressed child pathways are shown above the dotted line and colored by the following categories: immune regulation (blue), DNA replication and repair (yellow), wound healing (orange), small molecule biosynthesis (pink), muscle growth (purple), neuronal regulation (green), or other (gray). GO pathways listed below the plot are representative parent pathways for each color group. Pathway size is shown as bubble size (inset key). (**D**) Enrichment plots (left) and count heatmaps (right) of inset labeled pathways from panel **C**. Log base 2 fold change between I-*wt* and I-*ko* is shown in blue to green on the left, while normalized read counts for each group are shown as two columns to the right in purple. Significantly enriched genes as determined by DESeq2 are boxed. Five leading edge genes of each pathway that are either significantly enriched (boxed) and/or show the greatest enrichment are shown. Significantly enriched genes shown here are also labeled in panel **B**. Note that gene *Lep* is listed two times as part of two different pathways. Dotted lines in all plots represent *P* value cutoff of 0.05.

To identify host responses that account for the differences in urine UPEC titers between *Slpi^+/+^* and *Slpi^−/−^* mice, we performed bulk RNA sequencing on whole bladders. We included four groups of mice: (i) *Slpi^+/+^* mice undergoing mock-infection (M-*wt*), (ii) *Slpi^+/+^* mice infected with UTI89 (I-*wt*), (iii) *Slpi^−/−^* mice undergoing mock infection (M-*ko*), and (iv) *Slpi^−/−^* mice infected with UTI89 (I-*ko*). Mice were sacrificed and bladder tissues were harvested for RNAseq 1 day after inoculation (Fig. 4A).

We first contrasted infected I*-wt* and I*-ko* mice to their respective mock-infected control groups (M-*wt*, M-*ko*) to identify pathways significantly regulated by UTI. As expected, we found that infection triggered significant differential expression of 1,878 genes in I-*wt* compared to M-*wt,* many of which are genes involved in the inflammatory response (Fig. S3A; see also Table S1A). In *Slpi^−/^*^−^ mice, only 257 genes were differentially expressed between I*-ko* and M*-ko* mice, of which 212 were shared with infected *Slpi^+/+^* mice (Table S1B). Interestingly, despite these differences in gene-level regulation, 71% of the pathways significantly regulated by infection were shared between *Slpi^+/+^* (I*-wt* vs M*-wt*) and *Slpi^−/^*^−^ (I*-ko* vs M*-ko*) mice, suggesting that *Slpi^−/−^* mice respond to a UTI similarly to *Slpi^+/+^* (Fig. S3A; see also Table S2A and S2B). To understand these contrasting findings, we examined pathways expected to be upregulated during a UTI that were also shared between infected *Slpi^+/+^* (I*-wt* vs M*-wt*) and *Slpi^−/−^* (I*-ko* vs M*-ko*) mice (Fig. S3B). While the enrichment scores were similar between *Slpi^+/+^* (I*-wt* vs M*-wt*) and *Slpi^−/−^* (I*-ko* vs M*-ko*) groups, we found that many of the genes within these pathways were expressed more highly and these genes were more likely to be statistically significant in *Slpi^+/+^* (I*-wt* vs M*-wt*) mice (Fig. S3C). These data show that while *Slpi^−/−^* mice respond to infection similarly to *Slpi^+/+^* mice at the pathway level, they significantly upregulated fewer individual inflammatory genes and appeared to have a muted inflammatory response at 1 dpi compared to wild-type controls.

To identify genes regulated by the presence of SLPI, we first compared mock-infected *Slpi^−/^*^−^ and *Slpi^+/+^* mice (M-*ko* vs M-*wt*) which demonstrated few statistically significant differences at both the gene and pathway levels implying that in the absence of infection the transcriptional profiles of the bladder are very similar (Tables S1C and S2C). We next contrasted I-*ko* to I-*wt* mice to investigate the impact of SLPI on the transcriptional response to UTI. We found that there were 42 differentially expressed genes, most of which were only modestly altered in expression (log2 fold change <1; Fig. 4B; Fig. S3D; see also Table S1D). We additionally verified by qRT-PCR that several inflammatory genes known to be important in UTI were not different between *Slpi^+/+^* and *Slpi^−/−^* mice at multiple time points (Fig. S4A through C). However, pathway level analysis demonstrated 553 differentially expressed pathways, of which 508 were enriched in I-*wt* mice (Fig. 4C; see also Table S2D). Most of the pathways differentially enriched in I-*wt* mice are involved in immune regulation, DNA replication and repair, small molecule biosynthesis, and wound healing (Fig. 4C). These pathways encompass many of the genes found to be differentially expressed between I-*ko* to I-*wt* mice (Fig. 4D). Taken together, our findings suggest that the increased urine titers of UTI89 observed in *Slpi^−/−^* mice could be the result of a dysregulated immune response and delayed epithelial healing.

### *Slpi^−/−^* mice experience prolonged bladder inflammation after UTI

**Fig 5:**
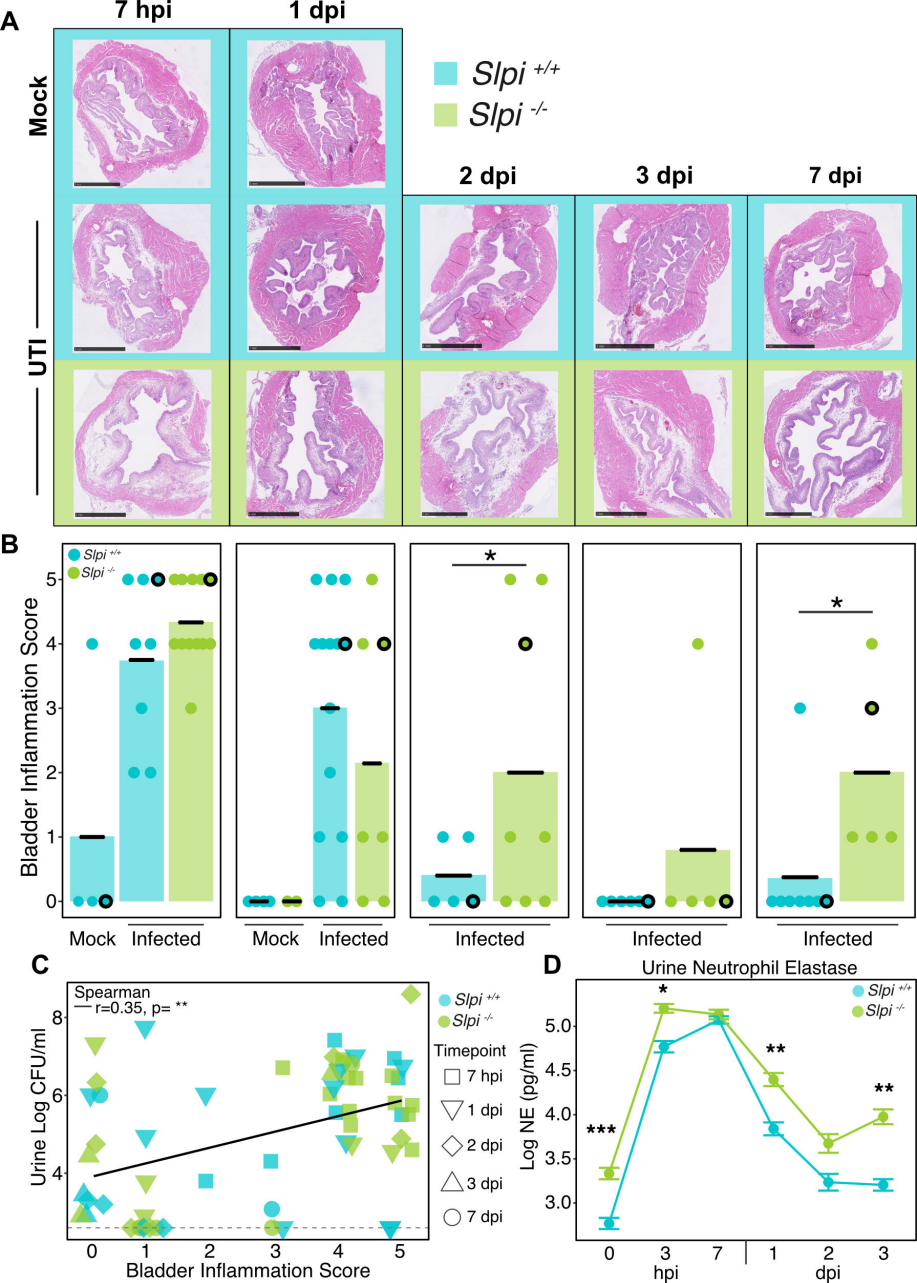
*Slpi^-/-^* mice experience prolonged bladder inflammation after UTI. (**A**) Representative hematoxylin and eosin staining of bladders from mock-infected *Slpi^+/+^* (blue, top row), UTI89-infected *Slpi^+/+^* (blue, second row), and UTI89-infected *Slpi^−/−^* mice (green, last row) at 7 hpi, 1, 2, 3, and 7 dpi. Images are 20× magnification with 1 mm scale bars. (**B**) Bladder inflammation scores (*n* = 4–14) mice per group, combined from five experiments (one-sided Student’s *t* test). Outlined points correspond to bladder images shown in panel A. (**C**) Correlation of bladder inflammation score to urine log base 10 CFU/mL for all time points (*n* = 2–12 per genotype per time point combined from five experiments; Spearman rank). (**D**) Log base 10 transformed neutrophil elastase (NE) levels in *Slpi^+/+^* (blue) and *Slpi^−/−^* mice (green) following infection. These data are combined from six experiments with 10–30 mice per genotype per timepoint (Student’s *t*-test).

We next asked whether the absence of SLPI was associated with delayed resolution of bladder inflammation following UTI. We assessed this by collecting urines and bladders from *Slpi^+/+^* and *Slpi^−/−^* mice over a period of 7 hours to 7 days. Hematoxylin and eosin (H&E) stained bladder sections were scored for inflammation severity on a scale of 0–5, where 0 was considered normal and 5 indicated severe damage to the uroepithelium (66). We found that *Slpi^−/−^* mice had significantly higher bladder inflammation scores at 2 (*P* = 0.04) and 7 dpi (*P* = 0.03), although whole bladder weights did not differ at 3 hpi (Fig. 5A and B; Fig. S4D). Overall, there was a positive correlation between inflammation score and urine UPEC titers as expected for *Slpi^+/+^* and *Slpi^−/−^* mice (*P* = 0.005; Fig. 5C). Finally, we measured NE levels in the urine of mice infected with UPEC. We found NE in urine from infected *Slpi^−/−^* mice was significantly higher than infected *Slpi^+/+^* mice prior to infection (*P* = 0.0003), and at both early (3 hpi, *P* = 0.01) and late time points (1 dpi, *P* = 0.002; 3 dpi, *P* = 0.008) (Fig. 5D), suggesting that SLPI may reduce the secretion and/or persistence of NE within the urinary tract even before infection.

### Levels of urine SLPI are changed in women with bacteriuria

Our mouse studies suggest that SLPI is increased in urine in response to UPEC-caused UTI, but the behavior of SLPI in the human urinary tract during UTI is not currently known. We addressed this gap in knowledge by obtaining urine and clinical information from female subjects 18–49 years old who had urine samples submitted to the clinical microbiology lab at Barnes-Jewish Hospital in St. Louis, MO. After collection, SLPI and total protein were quantified and correlated to the available clinical data.

**Fig 6:**
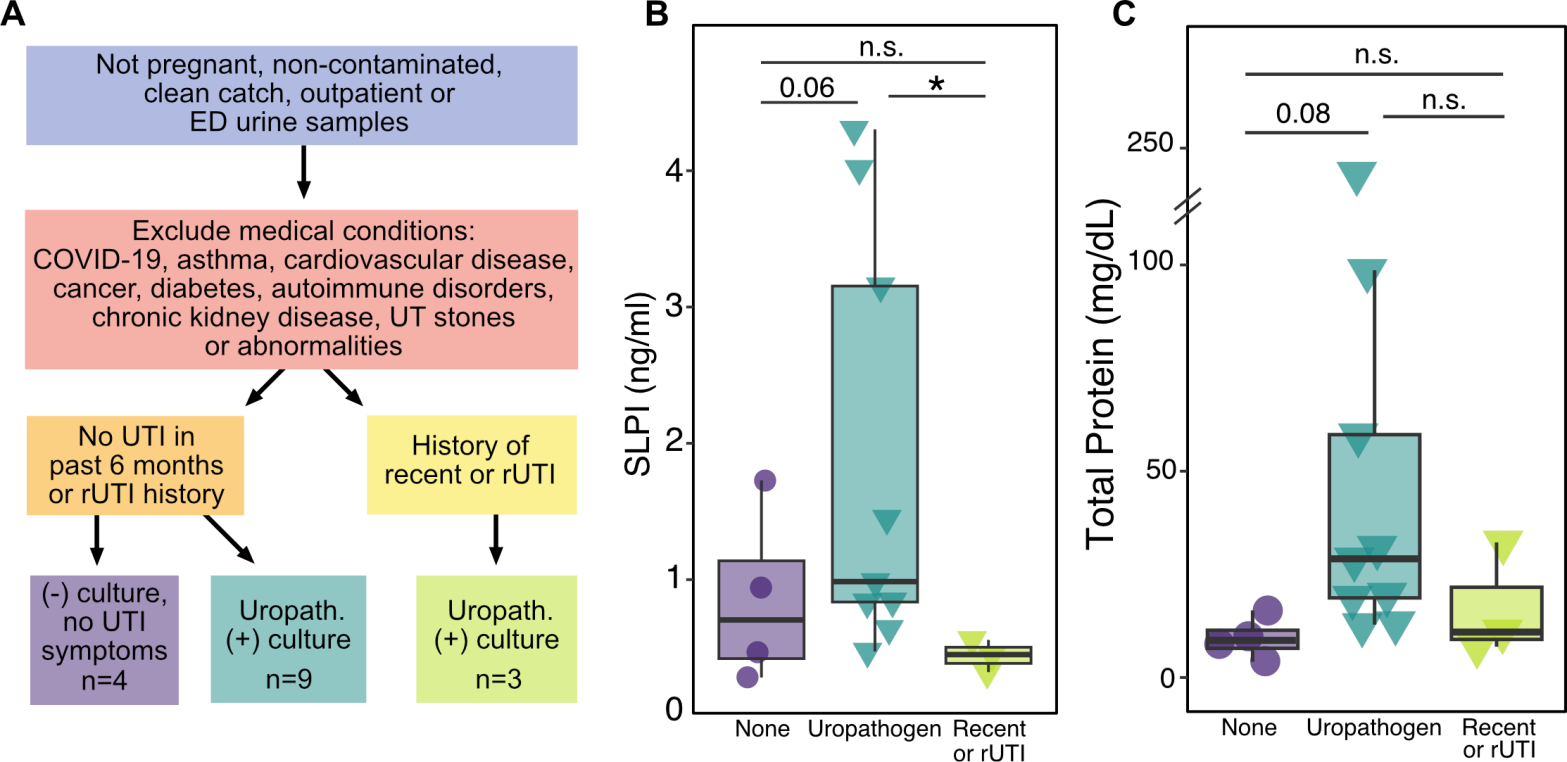
Levels of urine SLPI are changed in women with bacteriuria. (**A**) Flowchart describing the filtering process for clinical study patient samples. (**B**) SLPI protein measured in urine samples (one-sided Student’s *t* test). (**C**) Total protein in urine corresponding to samples in panel **B** (Student’s *t* test). Shapes represent urine culture status: negative culture with no UTI symptoms (circles), culture positive for uropathogen (triangle). All positive uropathogen cultures were positive for *Escherichia coli*.

We first excluded samples collected from pregnant individuals as well as samples from inpatient facilities to avoid potential confounding medical conditions (see Materials and Methods). We also excluded samples that were contaminated or collected by urinary catheters. Of the remaining samples, we then removed samples from subjects with comorbidities that could influence urine SLPI levels including COVID-19, asthma, cardiovascular disease, cancer, diabetes, autoimmune disease, chronic kidney disease, and urinary anatomic abnormalities (including nephrolithiasis). The remaining 16 samples were then divided into three groups: (i) the first group had cultures negative for a uropathogen, no current UTI symptoms, and no reported history of a recent UTI within the last 6 months or rUTI (>2 infections in the last month and/or >4 infections in any year prior); (ii) the second group had cultures positive for a uropathogen (defined here as >1 × 10^5^ CFU/mL) and no recent history of UTI or rUTI; and (iii) the third group had cultures positive for a uropathogen and a recent history of UTI or rUTI (Fig. 6A). A one-way ANOVA showed these groups did not differ in age (*P* = 0.9) or BMI (*P* = 0.5) (Table S3). Comparison of these three groups showed that individuals with a culture positive for a uropathogen without a history of rUTI trended toward greater amounts of urine SLPI when compared to subjects with a negative culture (Fig. 6B, *P* = 0.06). However, women with a history of recent or rUTI with a culture positive for a uropathogen did not demonstrate any elevation in their urine SLPI levels compared to women without a cultured uropathogen. They also had significantly less urine SLPI when compared to uropathogen positive samples from women without a history of recent or rUTI (*P* = 0.02). Taken together, these results implicate SLPI in the response to uropathogen exposure, but imply that its levels may be modulated by repeated exposure. Similarly, the uropathogen-positive group trended higher in total urine protein compared to subjects with negative cultures, but this group was not significantly different from patients with a history of recent or rUTI (Fig. 6C). This suggests that SLPI may be upregulated along with other proteins in response to a bacterial insult, but non-specific elevations in SLPI due to immune cell recruitment or tissue damage cannot be excluded. We also examined available urinalysis results for these patients (Fig. S5A) which include biomarkers often used for the diagnosis of a UTI including urine nitrite, leukocyte esterase (LE), and white blood cell count. However, we did not find any significant correspondence between any of these biomarkers and SLPI (Fig. S5B through D).

## DISCUSSION

SLPI is known to provide several protective effects to the host and has been directly demonstrated to aid in wound healing (28) and limit deleterious inflammation (67–69). While changes in SLPI expression have been associated with infections at mucosal sites, the relevance of SLPI during experimental infection is largely unexplored. Here, we use a well-characterized animal model of UTI (70) to show that SLPI is rapidly increased in the urine in response to UPEC challenge. Using a previously described mouse deficient in SLPI (*Slpi^−/^*^−^) (28), we show that mice lacking SLPI are more susceptible to severe UTI, as evidenced by dysregulation of the inflammatory response, increases in urine bacterial titers, as well as more severe and prolonged bladder inflammation. We also find that *Slpi^−/−^* mice have increased urine NE at baseline and during infection which likely contributes to the delayed histological resolution of infection. Our findings define SLPI as an important innate host factor that helps to repel bacterial pathogens within the urogenital tract, demonstrate a causal role for SLPI in resolving mucosal inflammation, and imply that SLPI may directly protect from infection at other mucosal sites.

SLPI is known to have immunomodulatory properties, including the ability to regulate the production of NFκB-derived inflammatory cytokines (58, 60, 71) and modulate neutrophil function (72, 73). Our observation that *Slpi^−/−^* mice demonstrate evidence of immune dysregulation at multiple stages of UPEC cystitis support an immune regulatory role for SLPI in the urinary tract. During the early stages of cystitis (7 hpi), we measured increased urine neutrophils in *Slpi^−/−^* mice but did not observe reduced bacterial titers at this same time point. At 1–2 dpi, *Slpi^−/−^* mice had reduced expression of inflammatory pathways compared to their *Slpi^+/+^* controls which was accompanied by increased urine titers of UPEC. Finally, during the resolution of infection 2 dpi and onward, microscopic evaluation of *Slpi^−/−^* mice showed delayed recovery of bladder epithelium from damage following infection. Taken together, these findings suggest that in the absence of SLPI, there is a dysregulated immune response that peaks earlier than in *Slpi^+/+^* mice and also fails to effectively resolve bladder inflammation. These findings are in line with previous studies showing that controlling UPEC in the bladder requires precise regulation of neutrophil recruitment to ensure adequate clearance of bacteria while avoiding excessive inflammation that can lead to mucosal damage and chronic infection (24). Better understanding of the multipurposed role for SLPI in regulating neutrophil function, immune signaling, and epithelial recovery will require future experiments to untangle the tissue-specific roles of SLPI in the bladder.

Additionally, our findings suggest that the immunomodulatory and anti-serine protease activities of SLPI are not mutually exclusive. One of the major biological functions of SLPI is to inhibit the activity of NE, a serine protease that promotes inflammation by degrading extracellular matrix proteins especially elastin (52, 53), causing increased permeability, and excessive tissue damage (52, 54–56, 74). During experimental UTI, SLPI-deficient mice had increased NE (but not neutrophils, Fig. 3B) in their urine even before infection and long after peak neutrophil infiltration in the urine, implying that SLPI limits NE abundance during homeostasis and infection in the urinary tract. This is potentially important because *Slpi^−/−^* mice had histologically more severe and prolonged bladder inflammation in our experiments, which could be explained by an overabundance of NE found in the bladder lumen. Taken together, these findings imply that SLPI, similar to the serpin antiprotease family (24), helps to resolve inflammation in the bladder following infection by limiting the mucosal damaging protease activity of NE (73) in the urinary tract.

While multiple cell types are known to express SLPI, epithelial cells are primarily responsible for producing and secreting SLPI at mucosal surfaces (41, 75, 76). However, many immune cell types including macrophages (77), neutrophils (72, 78), eosinophils (79), T (80), and B cells (81), express SLPI which may play a direct role in regulating immune cell function (50). In the urinary tract, SLPI likely originates from multiple cell types, including the bladder epithelium, and is also known to be directly filtered and secreted into the urine from the circulation through the kidneys (40, 82–84). Our study shows that SLPI is localized to bladder epithelial cells, suggesting urine SLPI is arising, at least in part, from the bladder epithelium during infection. It is not known if the presence of SLPI influences the exfoliation of the bladder epithelium during infection and increases in SLPI seen in the urine could come through enhanced exfoliation and lysis of infected cells in addition to increased secretion. Interestingly, rises in SLPI protein found in urine and cell culture supernatants following infection are not accompanied by corresponding increases in *Slpi* RNA transcripts. These findings raise the possibility that bladder cells contain a reservoir of SLPI that is rapidly released following pathogen challenge, or is post-transcriptionally regulated rather than transcriptionally regulated during the early stages of infection. Additionally, we cannot exclude the possibility that SLPI may also originate from recruited neutrophils and immune cells in our mouse model, though we note that neutrophil abundance in the urine typically subsides before SLPI protein levels return to baseline (85). Future experiments to assess differences in uroepithelial exfoliation and use of a tissue-specific deletion of SLPI will further elucidate the cellular origins of SLPI in the urinary tract during infection.

In addition to its antiprotease and immunomodulatory activities, SLPI has been reported to inhibit the growth of *E. coli* (47), *S. aureus* (47)*,* group A *Streptococcus* (86), and *P. aeruginosa* (64) in culture, and direct antimicrobial activity against UPEC would certainly explain our observation of increased urine titers in *Slpi^−/−^* mice during UTI. However, we did not detect bactericidal activity of commercially obtained recombinant SLPI (rSLPI) against the prototypical UPEC strain, UTI89, or a lab-adapted strain of *E. coli* in our *in vitro* studies. While we cannot exclude the possibility that SLPI inhibits UPEC growth under different *in vitro* conditions or only *in vivo*, cathelicidin (LL-37), another antimicrobial peptide found in the urinary tract (29), demonstrated high potency in the same assay. Our results are consistent with previous findings of *P. aeruginosa* infection of the airway (87) and suggest that the immunomodulatory functions of SLPI mediate its protective effects in our model of UTI.

We also investigated the role of SLPI in the human urinary tract by analyzing SLPI abundance in the urine of a cross-sectional group of women. When we focused on a subset of non-pregnant women without significant comorbidities (including prior UTI) that had negative urine bacterial growth or were positive for a uropathogen, we found SLPI tended to be increased in uropathogen-positive urine cultures. This finding supports our conclusions from our mouse experiments that SLPI initially increases rapidly in the urine in response to uropathogen invasion. However, we also noticed that women with a history of rUTI or recent UTI did not increase urine SLPI in the presence of a uropathogen. While this may appear to contradict our observations from our animal model, SLPI levels at other mucosal sites, like the lung, have been noted to decline in conditions of chronic inflammation and other antiproteases have been noted to have reduced expression in the bladder following rUTI (88, 89). Additionally, we note that decreases in urine SLPI have been described by other investigators in patients experiencing recurrent or frequent UTIs (84). These prior studies combined with our current findings suggest that SLPI may be rapidly released from bladders that are naive to infections, but that SLPI levels may decline with repeated or prolonged inflammation. This could be potentially explained by a reservoir of SLPI residing in the bladder epithelium that is rapidly released during an acute infection but is depleted with recurrent stimulation. Our immunohistochemistry (Fig. 2) supports this idea, showing that staining for SLPI within the bladder epithelium appears to be diminished in mice after experiencing UTI despite high levels of SLPI in the urine. Future studies dissecting the temporal relationship of urine SLPI to UTI, particularly in the context of recurrent UTI, will be necessary to untangle the relationship between SLPI, UTI, and chronic inflammation.

There are several limitations to this study that should be considered. First, while the *Slpi^−/−^* mice used in our study were backcrossed 10+ generations, we cannot exclude the possibility that passenger mutations (90) affecting genes in regions flanking *Slpi* (e.g. *Serinc3* and *Pkig*) indirectly influence our phenotypes. Reassuringly, in a reanalysis of previously published data, we found that many of the genes that were differentially expressed during UTI were also regulated by SLPI when it was over expressed in mouse preosteoblast cells (91) (Fig. S6), suggesting that our findings are independent of potential confounding passenger mutations. Second, in our clinical study, we do not have data about whether patients were treated for UTI, so we are uncertain if subjects with a cultured uropathogen were ultimately diagnosed with a UTI. Third, the subject population tended to have multiple comorbidities in addition to UTI and samples were collected during periods of high community transmission of COVID-19 infection. Additionally, approximately half of our samples were made up of routine urine screenings for pregnant women which were excluded from our analyses of SLPI and uropathogens (see Materials and Methods). As a result, the study population on which we could perform analyses was significantly reduced.

In summary, the early innate immune response to bacterial pathogens can set the stage for progression, persistence, or resolution of infection. Our findings show that SLPI restrains pathogen levels while simultaneously limiting inflammation, helping to achieve a delicate balance between the immune response and protection of the bladder tissues. We suggest that further understanding of the diverse roles SLPI plays in the urinary tract could improve our understanding and treatment of UTIs.

## MATERIALS AND METHODS

### Mice

All animal procedures were reviewed by the Washington University Institutional Animal Care and Use Committee (Protocol#: 21-0394). Female WT C57BL/6J mice were purchased from Jackson Laboratories (CAS: 00064, Bar Harbor, ME). Cryo-recovered 129;BL/6 SLPI^tm1Smw^/J mice (CAS: 010926, Jackson Laboratories, Bar Harbor, ME) were backcrossed to WT C57BL/6J mice for at least 10 generations before being used in these experiments. Female *Slpi^+/+^* and *Slpi^−/−^* mice were generated through homozygous breeding.

### Mouse urinary tract infection

A kanamycin-resistant derivative of a human cystitis isolate, UTI89*att_HK202_::KanR* (called UTI89 throughout this manuscript) and used to infect mouse bladders (61). This strain was a generous gift from Fimbrion Therapeutics, Inc. (St. Louis, MO) and was generated by inserting a kanamycin resistance cassette into the HK022 phage bacterial attachment site of the human cystitis UPEC isolate UTI89 (12) using the lambda red recombinase system, as previously described (92, 93). The insertion cassette was amplified from pKD4 using primers HK-FT-F and HK-FT-R, producing a linear PCR product with ends that are homologous to HK site sequences (Table S4A). The linear PCR product was then electroporated into UTI89 containing a temperature-sensitive helper plasmid pKM208 and plated on agar containing kanamycin. Recombinants were confirmed by PCR using flanking primers HK-test-Left and HK-test-Right to confirm insertion at the HK site (Table S4A).

Bacterial cultures were grown under type 1 pilus-inducing conditions. Briefly, 20 mL of Luria-Bertani broth (LB) was seeded from a glycerol stock and grown statically at 37°C overnight, then subcultured 1:1,000 in fresh LB and grown statically at 37°C for an additional 18–24 h. Cultures were centrifuged (1,750 × *g* for 15 min) and resuspended in sterile 1× PBS. Inoculum was diluted to a final concentration of 1–2 × 10^9^ colony forming units (CFUs)/mL (1:10 OD_600_ = 0.22–0.24) and 50 µL (~0.8–1 × 10^8^ CFU/mL) was used to inoculate the bladders of 6- to 8-week-old female mice. Transurethral catheterization was performed while mice were anesthetized using inhaled 2.5% isoflurane. Mock-infected mice were inoculated with sterile PBS in the same manner.

### Bacterial enumeration in mouse tissues

Following infection, mice were euthanized before bladder and kidneys were aseptically harvested into pre-weighed tubes containing 1 mL of sterile PBS. In some experiments, bladders were cut in half longitudinally with one section being saved for other assays. Final weights were recorded before homogenizing tissues. Samples (including urine) were serially diluted 1:10 in sterile PBS and 5 µL of each dilution was spotted onto LB agar supplemented with 50 µg/mL kanamycin (LB/Kan50) five times. Colony counts were averaged for the highest dilution containing colonies in four or more replicates and CFU/mL was calculated. This was normalized to total tissue weight to obtain CFU/g.

### *Ex vivo* gentamicin protection assay

An *ex vivo* gentamicin protection assay (63) was used to determine the amount of attached and intracellular UPEC with some modifications. Briefly, after 3 h of infection, half bladders were removed from mice and washed three times with 500 µL sterile PBS with the second wash used to quantify luminal UPEC. Bladders were then placed in PBS with gentamicin (100 µg/mL) and incubated at 37°C for 90 min. After washing with 1 mL of PBS, bladders were homogenized in 0.1% triton/PBS, diluted, and plated on LB/Kan50 agar plates to determine the amount of intracellular UPEC.

### Neutrophil quantification by cytospin

Urine was diluted 1:10 and 80 µL were spun onto poly-L-lysine-coated glass slides (750 × *g*, 10 min). Slides were air-dried, fixed for at least 1 h, and stained using the Shandon KwikDiff staining kit (CAS: 99-907-01, Thermofisher). Coverslips were mounted using Cytoseal XYZ (CAS: 22-050-262, Thermofisher) and allowed to dry. Investigator was blinded to slides before neutrophils were counted across three high power fields (HPF; 40× magnification) and then averaged for each sample.

### Protein quantification by ELISA

Urine and 5637 cell culture supernatant samples were thawed and diluted 1:10 (uninfected and mock-infected) or 1:50–1:1,000 (infected) in sterile PBS + 1% BSA. SLPI and NE were measured using the Mouse SLPI DuoSet ELISA (CAS: DY1735-05, R&D Systems) and Mouse Neutrophil Elastase (ELA2) Duoset ELISA (CAS: DY4517-05, R&D Systems) respectively, both according to the manufacturer’s instructions.

### Bacterial killing assay

UTI89 and DH5α were each shaken at 37°C in 5 mL of LB broth and then subcultured into a fresh 5 mL of LB. This culture was grown to log phase (~2 h) before being centrifuged (1,750 × *g*, 15 min), washed once with sterile 1× PBS, and resuspended to 1:10 OD_600_ = 0.15–0.2. The culture was then diluted 1:50 in 1× PBS + 1% LB broth and incubated statically at 37°C with recombinant human SLPI protein (rSLPI) (CAS: 1274-PI-100, R&D Systems) at these final concentrations: 20, 10, and 1 µM. A 1 µM concentration of LL-37 (CAS: tlrl-l37, Invivogen), the active portion of cathelicidin, was used as a positive control for bactericidal activity. Cultures were sampled after 2 h of incubation and titered as previously described.

### Bladder histological scoring

Half bladders were stored in 4% paraformaldehyde/water at 4°C overnight. The following day, samples were washed three times with 70% ethanol and then stored at 4°C until further processing. Samples were pinned to silicone plates, embedded in 1% agar, and stored in 70% ethanol until paraffin embedding and H&E staining was performed. Slides were then imaged using a Hamamatsu Nanozoomer at 20× power. An investigator, who was blinded to timepoints and experimental groups, assigned a score of 0–5 to each bladder using a grading system adapted to the robust UTI response seen in C57BL/6 mice (19). Scores used in this paper are defined as the following: 0, normal; 1, focal neutrophil infiltration; 2, diffuse neutrophil infiltration; 3, diffuse neutrophil infiltration and edema for early acute cystitis; 4, all of three plus neutrophils in muscle tissue; and 5, erosion of bladder epithelium.

### Bladder immunofluorescent staining

Bladders were harvested and processed for histology as described above. Unstained paraffin-embedded sections underwent paraffin removal using 100% xylenes, followed by 100% ethanol to remove excess xylene. Subsequent 5-min incubations in decreasing concentrations of ethanol (95%, 90%, 70%, and 50%) were used to rehydrate the tissue followed by water to remove residual ethanol. For antigen retrieval, a 10 mM sodium citrate + 0.05% Tween 20, pH 6.0 solution was used in which slides were microwave boiled three times and then left in the warm buffer for 30 min before being washed 3× in water. Samples were permeabilized for 10 min with 0.1% Triton-X100 in PBS, washed 3× with PBS, incubated 30 min with 0.1% TrueBlack Plus Lipofuscin Autofluorescence Quencher (CAS: 23014, Biotium), and then washed 3× with PBS. Slides were blocked for 30 min with 2% BSA + 2% donkey serum in PBS and then washed 3× with PBS. Then, samples were incubated in 5 µg/mL polyclonal rabbit anti-mouse SLPI antibody (CAS: NBP1-76803, Novus Biologicals) or rabbit polyclonal IgG isotype control (CAS: NBP2-24891, Novus Biologicals) diluted in PBS + 1% normal donkey serum (CAS: 017-000-121, Jackson ImmunoResearch) overnight at 4°C. The following day, samples were washed 3× in PBS before incubating for 2 h at room temperature in 2 µg/mL donkey anti-rabbit IgG conjugated to AF594 (CAS: A21207, Thermofisher). After three washes in PBS, a 5 µg/mL of Hoechst 33342 stain (CAS: H21492, Invitrogen) in PBS was added before a final three washes with PBS and coverslip mounting using ProLong Diamond Antifade Mountant (CAS: P36961, Thermofisher). Slides were imaged at 5× and 20× using a Zeiss Axio Observer Microscope and fluorescence display settings were determined using isotype and infected *Slpi^−/−^* mouse controls.

### Bladder RNA isolation

Half bladder samples were stored in 500 µL of RNAlater (CAS: AM7021, Thermofisher) and incubated overnight at 4°C. The following day, RNAlater was removed and samples were transferred to −80°C until further processing. To isolate RNA, bladders were thawed, transferred to 1 mL of TRIzol, and homogenized. A 0.3-mL portion of this homogenate was used to extract and purify RNA using the RNeasy Mini Kit (CAS: 74104, Qiagen) following the manufacturer’s instructions, then eluted in 25 µL of RNAse-free water.

### Quantitative PCR on bladder homogenate

After extraction, RNA quality was checked by gel electrophoresis and quantified using Ribogreen (CAS: R11490, Invitrogen). High-Capacity cDNA Reverse Transcription Kit (CAS: 4387406, Applied Biosystems) was used to reverse transcribe 100 ng of RNA to cDNA according to the manufacturer’s instructions. cDNA was diluted 1:3 or 1:4 before mixing with Power SYBR Green PCR Master Mix (CAS: 4368702, Applied Biosystems) and primer pairs (Table S4B). Results were analyzed using the ddCT method (94).

### Bulk RNA sequencing on whole bladder

Following extraction, RNA concentration was determined using Qubit RNA Quantification Assay, High Sensitivity (CAS: Q32852, Invitrogen) before assessing RNA quality by BioAnalyzer (Agilent) to ensure all samples had a minimum RNA integrity number (RIN) of 8. Stranded, poly-A enriched libraries were created using the NEBNext Poly(A) mRNA Magnetic Isolation Module (CAS: E7490S, NEB), followed by the NEBNext Ultra II Directional RNA Library Prep Kit (CAS: E7760S, NEB). Completed libraries were then sequenced to an average depth of approximately 20M reads per sample on a partial lane of the NovaSeq6000 S4 XP flow cell using 2 × 150 paired-end reads with 10-base dual indexes (CAS: E6440S, NEB). After demultiplexing these samples, reads were mapped to the Ensembl release 109 cDNA database using Salmon with default parameters (95). Differentially expressed genes were identified using DESeq2 (v1.34.0) (96) and then mapped to entrez ID using biomaRt (v2.50.3) (97). Functional pathway analysis was performed using fgsea (v1.20.0) (98) and mouse biological process GO database to determine significantly altered pathways between groups.

### Bladder cell culture

Human bladder epithelial cells (5637) were maintained in RPMI 1640 (CAS: 11875085, Gibco) with 10% FBS (CAS: 16000-014, Gibco) in a humidified chamber set at 37°C with 5% CO_2_. Bacterial cultures for these experiments were grown for type 1 pilus expression as described above. The day prior to infection, 24-well plates were seeded with 2 × 10^5^ cells/well in 0.5 mL and incubated in the chamber overnight. The following day, 95–98% cell confluency was confirmed and media was replaced 3–4 h before adding 50 µL of bacteria (4–7 × 10^6^ CFU, MOI 18–30) or PBS, then plates were gently centrifuged to settle bacteria onto cells before incubating at 37°C with 5% CO_2_. A 50-µL sample of supernatant was removed at 0, 0.5, and 1 h of infection with the remaining supernatant collected at 2 h. At this time, cells were resuspended in TRIzol reagent and frozen at −20°C until RNA was extracted using the Macherey-Nagel Nucleospin RNA XS Kit (CAS: 740902.50, Takara Bio) according to the manufacturer’s instructions. A 250-ng input of RNA was used to make cDNA which was subsequently diluted 1:5 in nuclease-free water before being used in qPCR as described above.

### SLPI-UTI clinical study

This study was approved by the Institutional Review Board of Washington University in St. Louis (Protocol#: 202107050) which was granted a waiver of consent. It was designed to investigate the contribution of SLPI in urinary tract infection. Leftover urine used in this study was sourced from original samples submitted to a clinical microbiology lab for routine culturing and/or urinalysis testing and consent was waived. Inclusion criteria required St. Louis Barnes Jewish or Children’s Hospital patients to be female and 18–49 years old. Samples were collected between August 2021 and February 2022. Urine was aliquoted in 1 mL portions and stored at −80°C until further processing. SLPI was measured in urine samples diluted 1:20–1:200 in sterile PBS + 1% BSA using the Human SLPI DuoSet ELISA kit (CAS: DY1274-05, R&D Systems) according to the manufacturer’s instructions. Total protein in the urine was measured using Quantichrom Total Protein Assay Kit (CAS: QTPR-100, BioAssay Systems) according to the manufacturer’s instructions with samples detected at a 1:4–1:20 dilution. A total of 202 patient samples were collected of which total protein and SLPI were measured in 197 samples. We defined a recent UTI as a documented infection within the last 6 months. rUTI was defined as more than two infections within the month before sampling and/or more than four infections within any year prior.

We noted that pregnant women, who constituted 48.7% of the available urine samples, had significantly increased levels of SLPI in their urine compared to non-pregnant women (Table S2, Figure S7A). Given these differences, we excluded pregnant women from our analysis of uropathogens and SLPI.

### Statistics

Statistical analysis was performed using R Version 4.1.2 (R Development Core Team, 2021). Data are presented as mean with error bars denoting SEM, box and whisker plots, or scatterplots. Log_10_ transformed data prior to mean and SEM calculations are indicated in the figure axis. Unless otherwise stated, statistical significance was conducted using Student’s *t* test. Boxplots show interquartile range (IQR) and whiskers display 1.5*IQR. Time series ANOVA was performed using Amelia (v1.8.0) package in R to impute missing data, followed by Student’s *t* test with FDR correction. Associations were determined using Spearman’s Rank. In all figures, the following symbols were used to designate significance: n.s. = not significant, *P < 0.05, **P < 0.01, ***P < 0.001, and ****P < 0.0001.

## Data Availability

RNAseq data have been deposited at the European Nucleotide Archive (https://www.ebi.ac.uk/ena/browser/home) and are publicly available under project accession number PRJEB66138.

## SUPPLEMENTAL MATERIAL

Supplemental Figures mbio.02554-23-s0001.pdf Figures S1-S7.

Table S1 mbio.02554-23-s0002.xlsx Differential gene expression.

Table S2 mbio.02554-23-s0003.xlsx Gene set enrichment analysis.

Table S3 mbio.02554-23-s0004.xlsx Clinical study demographics.

Table S4 mbio.02554-23-s0005.xlsx Primers.

ASM does not own the copyrights to Supplemental Material that may be linked to, or accessed through, an article. The authors have granted ASM a non-exclusive, world-wide license to publish the Supplemental Material files. Please contact the corresponding author directly for reuse.
