# Supplementary material for: Secretory leukocyte protease inhibitor protects against severe urinary tract infection in mice
Source: mBio. 2024 Jan 25;15(2):e02554-23. doi: 10.1128/mbio.02554-23 (PMC10865866; doi:10.1128/mbio.02554-23)
Supplement: Supplemental Figures — Figures S1-S7. [file mbio.02554-23-s0001.pdf]

Supplemental Figure 1

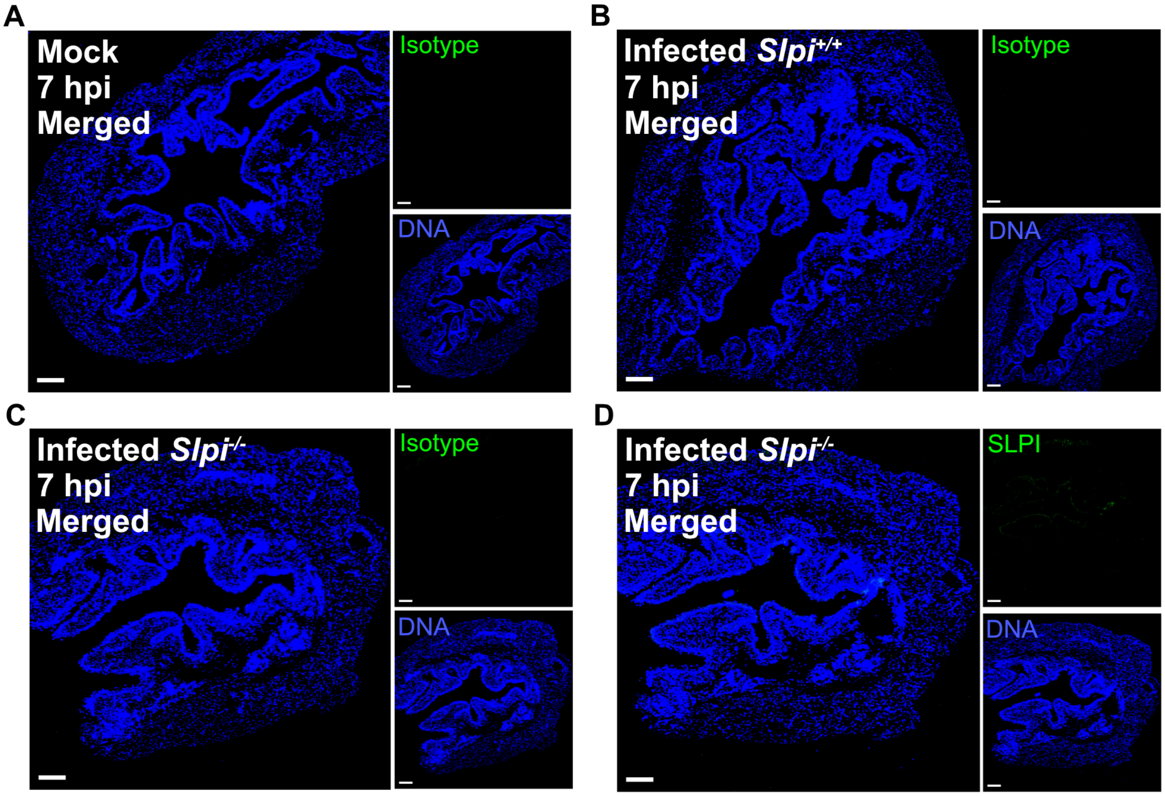

Figure S1: Immunofluorescent staining in the bladder is specific to SLPI
A) Mock-infected *Slpi*$^{+/+}$ mouse bladder stained with IgG isotype control antibody. Control staining on a serial section from bladder shown in Figure 2A. **B)** UTI89-infected *Slpi*$^{+/+}$ mouse bladder stained with IgG isotype control antibody. Control staining on a serial section from bladder in Figure 2B. **C)** UTI89-infected *Slpi*$^{-/-}$ mouse bladder stained with IgG isotype control antibody. **D)** UTI89-infected *Slpi*$^{-/-}$ mouse bladder stained with anti-SLPI antibody shown on a serial section of (C). All bladders were taken at the 7 hpi timepoint and stained for DNA. In each panel, the left image represents merged images of two right panels at 5X magnification with 200 µm scalebars.

Supplemental Figure 2

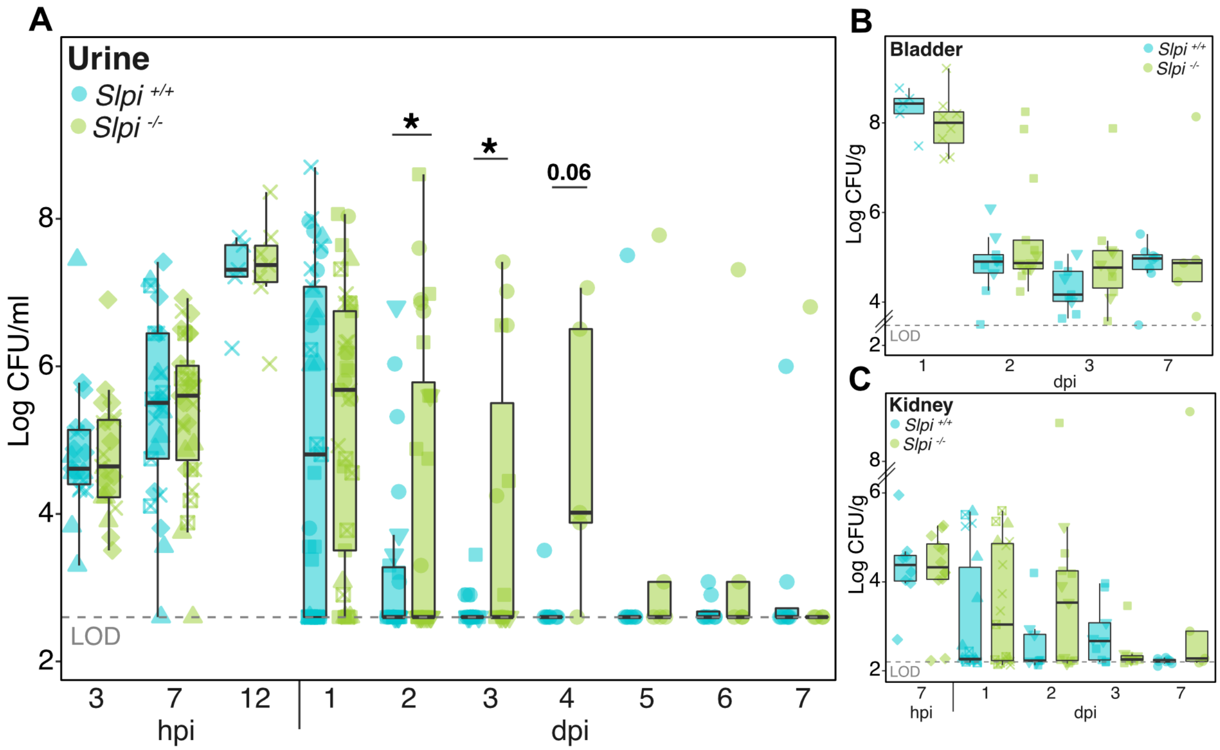

Figure S2: UTI89 bacterial titers shown with all datapoints
A) Log base 10 CFU/ml UTI89 in urine titers of *Slpi*$^{+/+}$ (blue) and *Slpi*$^{-/-}$ (green) over time. **B)** Log base 10 CFU/g UTI89 in bladder homogenates. **C)** Log base 10 CFU/g UTI89 in kidney homogenates. Shapes denote separate experiments and dashed gray lines show the limit of detection (LOD) for each tissue.

Supplemental Figure 3

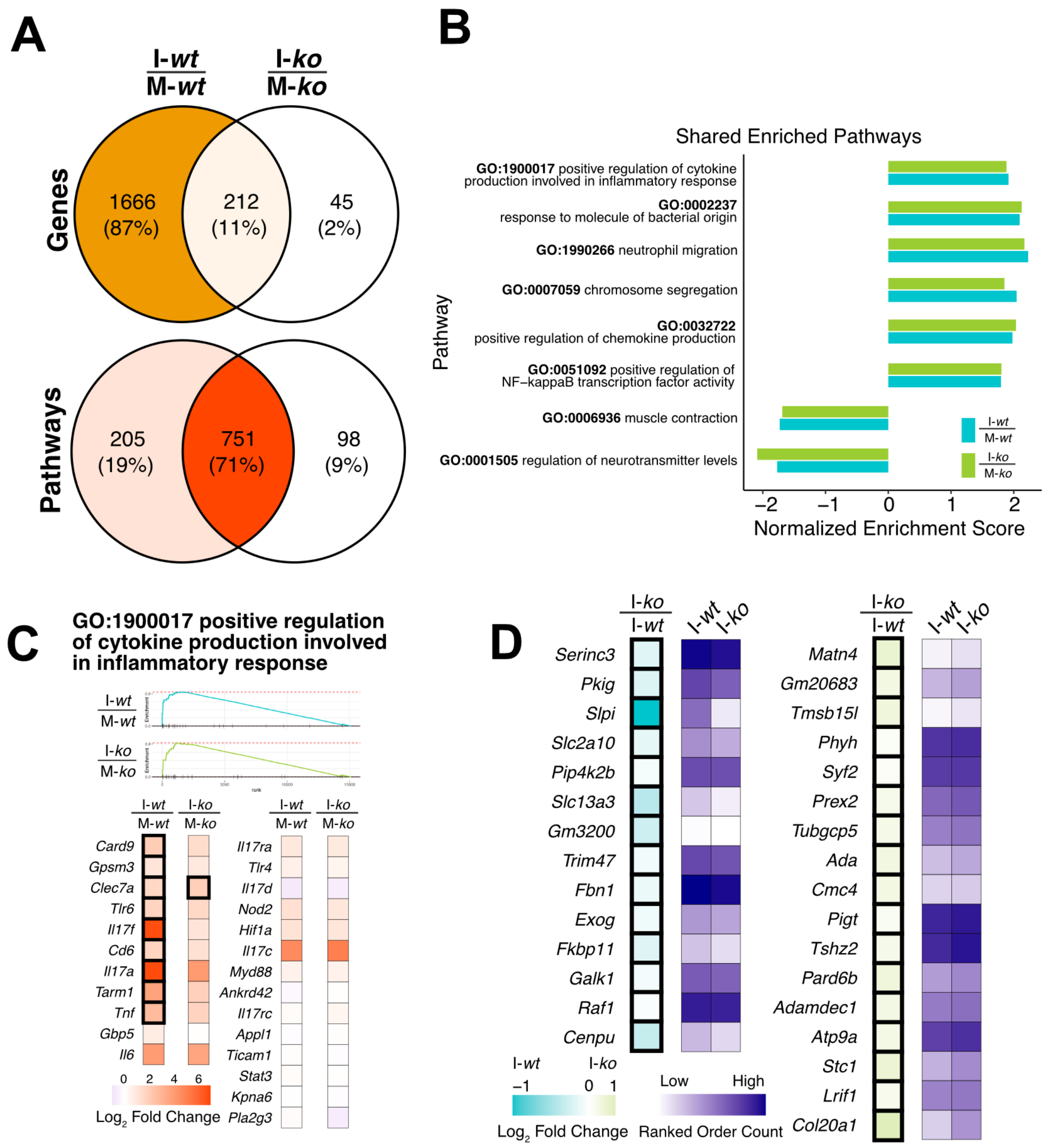

Figure S3: Transcriptional response *Slpi*$^{-/-}$ and *Slpi*$^{+/+}$ mice to UTI in the bladder at 1 dpi

A) Venn diagrams summarizing the differences in genes (top) and pathways (bottom) enrichment in *Slpi*$^{+/+}$ (I-*wt* vs M-*wt*) and *Slpi*$^{-/-}$ (I-*ko* vs M-*ko*) mice following infection. Infection significantly regulates a larger number of genes in *Slpi*$^{+/+}$ mice but the number of regulated pathways is similar in both groups. **B)** NES scores of select immune and UTI-related pathways significantly enriched in both *Slpi*$^{-/-}$ and *Slpi*$^{+/+}$ mice following infection. **C)** Enrichment plots (top) for GO:1900017 pathway from (B) for both I-*wt*/M-*wt*

(top) and I-*ko*/M-*ko* (bottom) comparisons. Heatmaps (lower panel) of log base 2 fold change (purple to orange) for all genes found in dataset for this pathway. Boxes indicate significantly enriched genes by DESeq2. Data illustrates fewer significantly regulated genes in *Slpi*$^{-/-}$ mice after infection despite a similar number of significantly regulated pathways. **D)** Heatmaps of log base 2 fold change (blue to green; first column) and ranked order of read counts (purple; second and third columns) of genes in Figure 4B that were significantly regulated but were not labeled. Left part of panel represents genes upregulated in I-*wt* mice while the right part of the panel are genes upregulated in I-*ko* mice.

Supplemental Figure 4

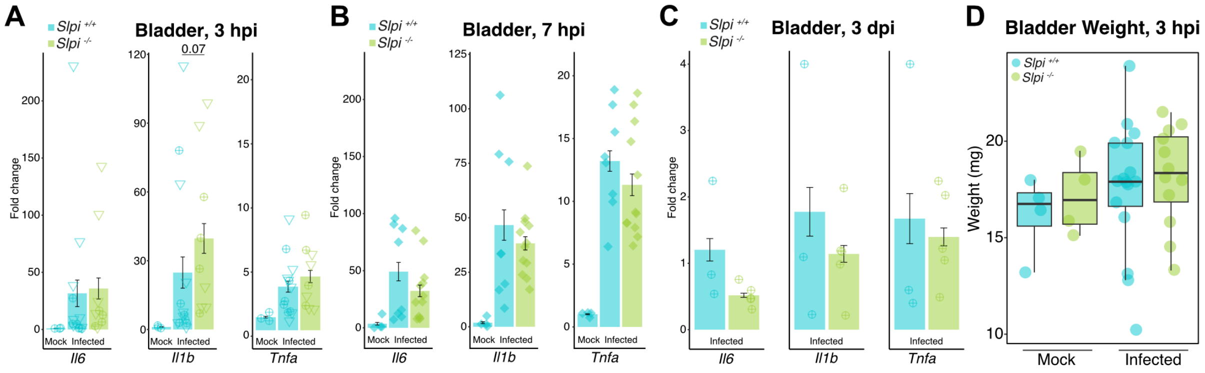

Figure S4: Markers of bladder inflammation following UTI in *Slpi*$^{+/+}$ (blue) and *Slpi*$^{-/-}$ mice

A-C) qRT-PCR of bladder from *Slpi*$^{+/+}$ (blue) and *Slpi*$^{-/-}$ (green) mice at (**A**) 3 hpi (n=9-13 mice per group) (**B**) 7 hpi (n=8-12 mice per group) and (**C**) 3dpi (n=3-5 mice per group). For each panel, fold change of NFκB-regulated inflammatory cytokines *Il6* (left), *Il1b* (middle), and *Tnfa* (right) are shown as fold change to either mock-infected *Slpi*$^{+/+}$ mice (panels (A) and (B); n=4) or as fold change from infected *Slpi*$^{+/+}$ mice (panel (C)). For all panels, each point represents one mouse and shapes denote 4 separate experiments (Student's t-test). **D)** Whole bladder weights from *Slpi*$^{+/+}$ (blue; n=15) and *Slpi*$^{-/-}$ (green; n=12) mice compared to mock-infected mice (n=4) combined from 2 separate experiments.

Supplemental Figure 5

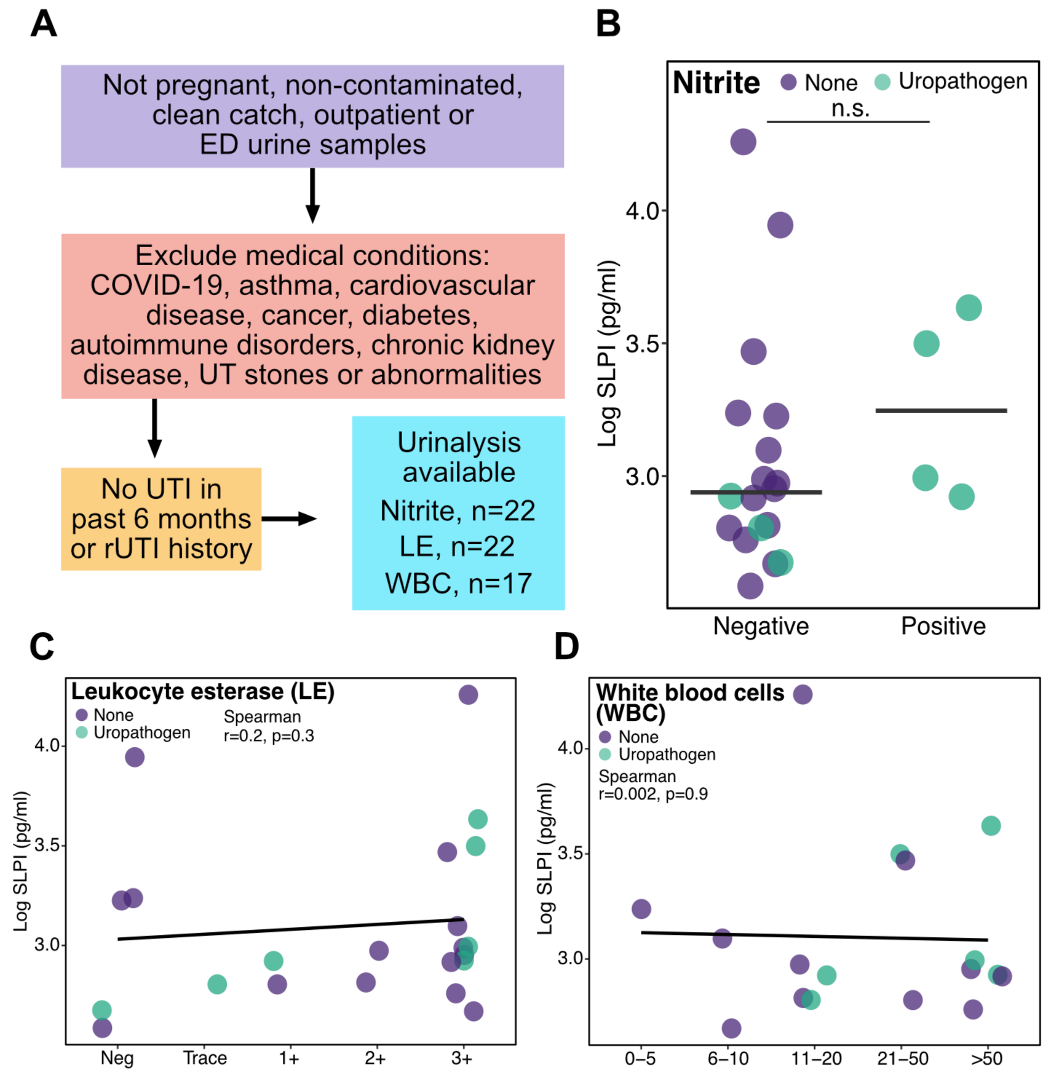

Figure S5: Relationship of urine SLPI to pregnancy and markers of UTI
A) Flowchart for available urinalysis results. **B)** Comparison of log base 10 transformed SLPI levels in nitrite positive and negative urine samples. **C)** Spearman correlation of log base 10 SLPI to leukocyte esterase. **D)** Spearman correlation of log base 10 SLPI to white blood cell count. Uropathogen positive samples are shown in teal and uropathogen negative samples are shown in purple in panels B-D.

Supplemental Figure 6

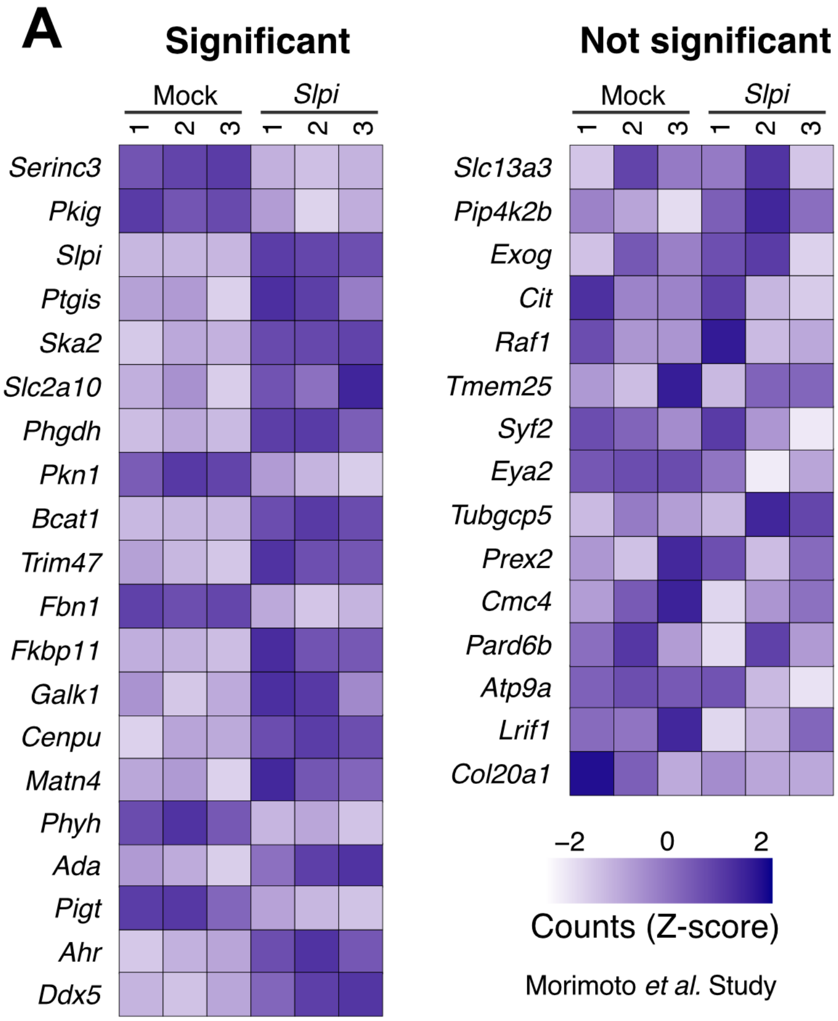

Figure S6: Comparison of genes differentially expressed by SLPI overexpression to those induced by UTI.
Data were accessed from Gene Expression Omnibus (GSE117532) and were previously published by Morimoto *et al.*[76]. Data is from RNAseq performed on mouse preosteoblasts and include cells over-expressing SLPI (n=3) and a mock control (n=3). Heatmaps show the z-score by row of raw counts. The left panel shows genes significantly regulated by overexpression of SLPI in preosteoblasts that were also found to be significant between I-*ko* and I-*wt* mice (Figure 4). The right panel shows genes that were not regulated by overexpression of SLPI in preosteoblasts that were significantly different in our study. Significance was determined by Student's t-test.

Supplemental Figure 7

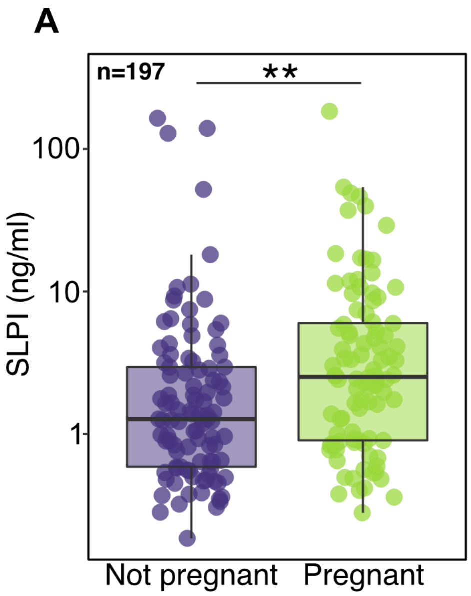

Figure S7: SLPI is increased in urine during pregnancy
A) Urine SLPI from pregnant (green, n=93) and not pregnant (purple, n=104) individuals (Student's t-test).
